# Letter from the Editor in Chief

**DOI:** 10.19102/icrm.2020.110805

**Published:** 2020-08-15

**Authors:** Moussa Mansour


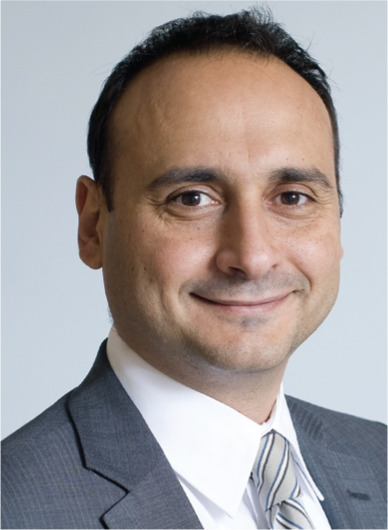


Dear Reader,

Atrial fibrillation (AF) affects a large number of patients in the United States and worldwide and presents a significant clinical and economic burden given its association with an increased risk of stroke, congestive heart failure, and premature death. Stroke in particular is the most devastating complication of AF: patients with AF are five times more likely to experience a stroke than those without. While anticoagulation therapy has been shown to be very effective in reducing stroke in AF, the effectiveness of this treatment modality has been limited by a high rate of nonadherence. In fact, according to one study, the rate of nonadherence to anticoagulation therapy at three years after initiation was more than 10% for direct oral anticoagulants and even higher for warfarin.^1^

Meanwhile, data from the PINNACLE study suggest that only 40% of patients who meet the criteria for anticoagulation are using such medications.^2^ Many factors have been implicated as forces driving this lack of widespread anticoagulation in patients with AF who are at risk of stroke, including the heightened risk of bleeding. However, many patients with a low risk of bleeding are also not receiving anticoagulation; in these cases, the patient’s lack of knowledge regarding the risk of stroke in AF may instead be playing a role. For many patients opting to not start an anticoagulant (passive risk-taking), such seems to be a safer course of action than that of beginning a blood thinner (active risk-taking), highlighting the importance of and need for patient education and engagement.

This issue of *The Journal of Innovations in Cardiac Rhythm Management* contains an important manuscript titled “Engaging Patients in Atrial Fibrillation Management via Digital Health Technology: The Impact of Tailored Messaging,” where Toscos et al. present the results of a randomized study comparing a combination patient management strategy encompassing the use of three digital health technologies (a patient portal, an electronic-prescribing data feed, and a smart pill bottle) with the conventional approach. Eighty patients were enrolled into each study group and, at six months of follow-up, patients in the intervention group showed greater AF knowledge. Along these lines, there was also a trend toward better adherence in the intervention group, although it did not reach statistical significance, likely because of the short duration of follow-up and small number of patients included. Despite these limitations, however, this study highlights the importance of adequate patient education and engagement, which I believe are critical for improving the outcomes of patients with AF.

I hope that you enjoy reading this article and the remaining papers in this issue.

Sincerely,


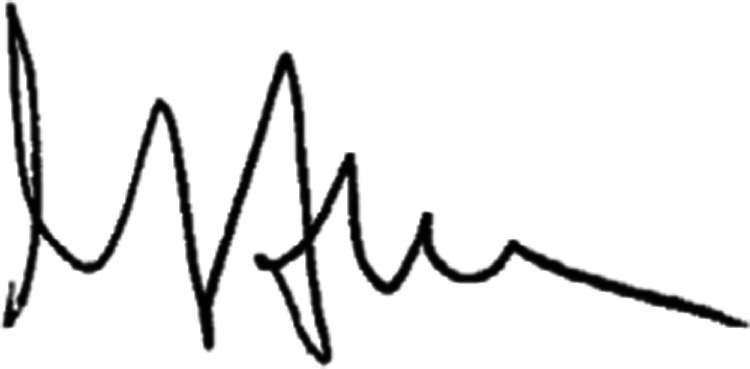


Moussa Mansour, MD, FHRS, FACC

Editor in Chief

The Journal of Innovations in Cardiac Rhythm Management

MMansour@InnovationsInCRM.com

Director, Atrial Fibrillation Program

Jeremy Ruskin and Dan Starks Endowed Chair in Cardiology

Massachusetts General Hospital

Boston, MA 02114
